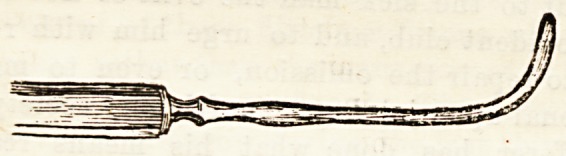# On Modern Progress in Ophthalmic Medicine and Surgery

**Published:** 1896-01-11

**Authors:** Robert Brudenell Carter

**Affiliations:** Consulting Ophthalmic Surgeon to St. George's Hospital.


### ON MODERN PROGRESS IN OPHTHALMIC MEDICINE AND SURGERY.

By ROBERT BRUDENELL CARTER, F.R.C.S., Consulting Ophthalmic Surgeon to St. George's Hospital.

SQUINT (*continued from page* 161).

THE uncertainty of the results attending the early operations for squint was not diminished until after 1860, and then in consequence of the discovery by Donders that the deviation is mostly a consequence of muscular efforts made to overcome an error of refraction. This discovery, which at once cleared away all the current hypotheses about the effects of imitation, or about disease of the motor apparatus, showed, at the same time, that it was often necessary to supplement tenotomy by the constant use of spectacles, and that, unless this were done, the persistence of the conditions by which the squint had been produced was very likely to lead to its return. In some instances it was found that spectacles alone would suffice to cure the deviation, or, what was more usual, would keep it in abeyance while they were worn. It soon became by no means uncommon to see spectacled children, whose eyes were to all appearance in correct position, but in whom, if the spectacles were removed, one eye instantly turned in.

The unsightly results of the operations first performed were not limited to occasional divergence or to occasional protrusion of the eyeball. It was almost universal for the caruncle of the affected eye to be drawn back within the canthus and concealed from view, so as to give the eye something of the appearance of an artificial one, an appearance heightened by the not uncommon limitation of its movements towards the side on which the tenotomy had been performed. The late Mr. Critchett, in this country, and the late Albrecht von Graefe, in Germany, applied themselves to the study of the anatomy of the eye muscles, and they both devised improved methods of operating, by which they hoped greatly to diminish the number of imperfect or untoward results. They both endeavoured to limit the effects of tenotomy by performing it with increased precautions against the division of tissues lying beyond the actual tendon, and they both succeeded to a considerable extent. Von Graefe made a small scissor-cut through the conjunctiva over the middle of the tendon, and then tunnelled downwards with the points of his scissors to a spot below the lower margin of the tendon, close to its insertion. At this spot he insinuated under the tendon the clumsy squint hook of those days (shown of natural size in the figure), carried it upwards until its point appeared beneath the conjunctiva above the upper margin of the tendon, and divided the included tissue with the scissors by a succession of little snips, keeping as close as possible to the eyeball. This was done without an anæsthetic; and, if the effect seemed to be greater than was desired, a fine suture was passed through the tendon and the conjunctiva, and was so tied as to bring the former somewhat back towards its original position. Mr. Critchett invented the method of subconjunctival tenotomy. He made a horizontal incision through the conjunctiva, parallel with and below the lower margin of the tendon, pushed his scissors upwards so as to detach the conjunctiva and subconjuctival tissue from the tendon in a narrow vertical track just over the insertion of the latter, then glided the hook under the tendon as a guide to one blade of the scissors, while the other blade was immediately under the conjunctiva, the tendon being included between the two. It could then be divided either by successive snips, or by one stroke of the scissors; and as soon as this was done the hook, no longer held back by the tendon, would break through the slender subjunctival tissue, and would advance right up to the margin of the cornea. As long as the hook was held back, it was certain that some portion of the tendon remained undivided, and that little or no effect upon the position of the eye would be produced.

Mr. Critchett's operation was in every way greatly superior to that of Von Graefe, and succeeded admirably in a large number of cases. But it had the disadvantage of looking very easy when it was performed by skilful hands. A distinguished professor of ophthalmology once told me that he had looked upon this operation as the easiest in surgery when he had only seen it done by others, but that, when he began to do it himself, he soon regarded it as one of the most difficult. The practised operator made his horizontal incision through the conjunctiva, of the necessary length and depth, by one closure of his scissors, cleared his subconjunctival track by another, rotated the eye a little inwards by fixation forceps so as to relax the muscle, under which the hook seemed almost to find its own way, and completed the operation by one or, at most, two scissor cuts. The tendon, still held by the sheath derived from the capsule of Tenon, could only retract in a moderate degree, and nothing but its attachment to the sclera was divided. The bungler had to dissect to find the tendon, and made indefinite excursions with his hook before he could succeed in securing it. Even then it sometimes escaped from him, and had to be recovered at the expense of more disturbance of the delicate tissues involved. It was often only partially divided at the first attempt, and its upper margin had to be sought for afterwards. The general result was, that it was not only severed from the sclera, but that it was also freely separated from its attachments in every direction, and was left free to retract even to the equator. The large, irregular, and lacerated wound was often troublesome in healing, and was apt to be the seat of a granulation which required to be snipped off and cauterised before the natural appearance was restored. With a skilfully performed operation the effect was often good and nearly always passable; but in unskilful hands the results usually left much to be desired. Some surgeons endeavoured to overcome the difficulties which they experienced by the invention of special instruments, but these were seldom found to be of material assistance. Even in the hands of the best operators, impaired mobility of the eye, or even relative divergence, were by no means impossible consequences of the methods of tenotomy which, until quite recently, have obtained.

## Figures and Tables

**Figure f1:**